# Synthesis, spectroscopic and crystal structure studies of *N*-{3-cyano-1-[2,6-di­chloro-4-(tri­fluoro­meth­yl)phen­yl]-4-(ethyl­sulfan­yl)-1*H*-pyrazol-5-yl}-2,2,2-tri­fluoro­acetamide

**DOI:** 10.1107/S2056989022009653

**Published:** 2022-10-04

**Authors:** Prabhakar Priyanka, Bidarur K. Jayanna, Yelekere C. Sunil Kumar, Mellekatte T. Shreenivas, Gejjelegere R. Srinivasa, Thayamma R. Divakara, Hemmige S. Yathirajan, Sean Parkin

**Affiliations:** a Department of Chemistry, B.N.M. Institute of Technology, Bengaluru-560 070, India; b Honeychem Pharma Research Pvt. Ltd., Peenya Industrial Area, Bengaluru-560 058, India; c T. John Institute of Technology, Bengaluru-560 083, India; d Department of Studies in Chemistry, University of Mysore, Manasagangotri, Mysuru-570 006, India; e Department of Chemistry, University of Kentucky, Lexington, KY, 40506-0055, USA; University of Neuchâtel, Switzerland

**Keywords:** crystal structure, phenyl­pyrazole, insecticide

## Abstract

The structure of the title compound, C_15_H_8_N_4_Cl_2_F_6_OS, a phenyl­pyrazole-based insecticide related to ethiprole, fipronil, and derivatives thereof is presented. The pyrazole ring has four chemically diverse substituents, namely a nitro­gen-bound 2,6-di­chloro-4-tri­fluoro­methyl­phenyl and carbon-bound cyano, ethyl­sulfanyl, and 2,2,2-tri­fluoro­acetamide groups. The pyrazole and phenyl rings are perpendicular, subtending a dihedral angle of 89.80 (5)°. In the crystal, strong N—H⋯O hydrogen bonds link the mol­ecules into chains that extend parallel to the *a*-axis.

## Abstract 2

The synthesis, crystal structure, and some spectroscopic details for the phenyl­pyrazole-based insecticide *N*-{3-cyano-1-[2,6-di­chloro-4- (tri­fluoro­meth­yl)phen­yl]-4-(ethyl­sulfan­yl)-1*H*-pyrazol-5-yl}-2,2,2- tri­fluoro­acetamide (C_15_H_8_N_4_Cl_2_F_6_OS) are presented.

## 1. Chemical context

The title compound is a phenyl­pyrazole-based insecticide. It is related to ethiprole, an insecticide used to kill or remove insects from crops and grains during storage (Arthur, 2002 ▸). Phenyl­pyrazole insecticides render an insect’s central nervous system toxic by blocking the body’s glutamate-gated chloride channel. Ethiprole itself is a non-systemic insecticide that is effective against a wide range of chewing and sucking insects (Wu, 1998 ▸) and is an active ingredient used in many insecticides for crop-protection products. Fipronil (see, for example, Park *et al.*, 2017 ▸) and fipronil sulfone belong to the same class of compounds. The design, synthesis, and insecticidal activity of novel phenyl­pyrazoles containing a 2,2,2-tri­chloro-1-alk­oxy­ethyl moiety have been published by Zhao *et al.* (2010 ▸). 
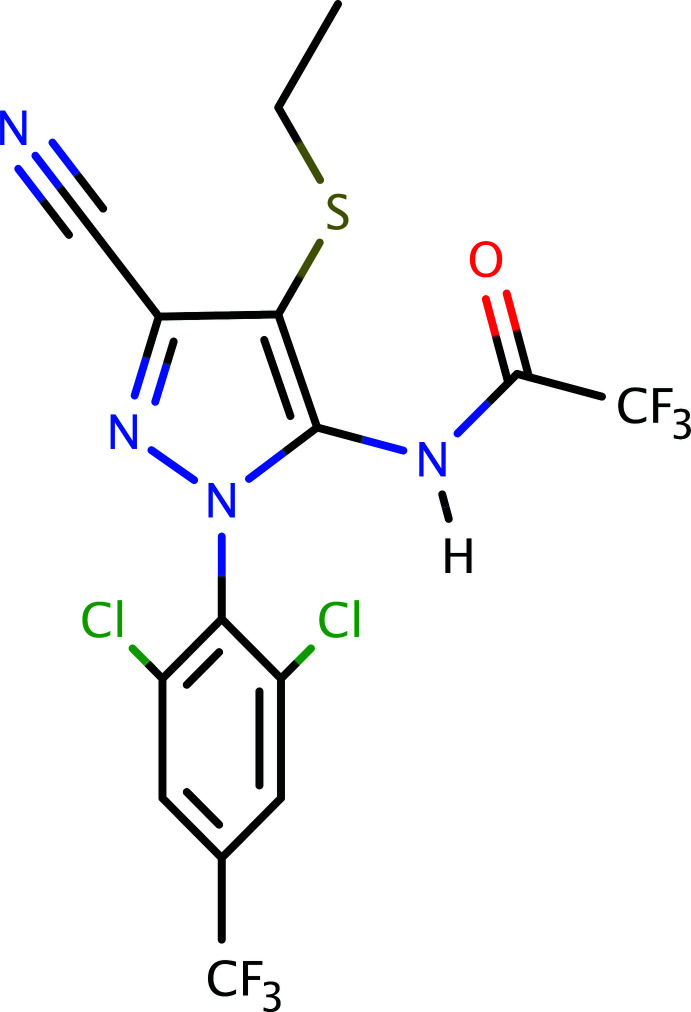


The starting material for the title compound, 5-amino-1-[2,6-di­chloro-4-(tri­fluoro­meth­yl)phen­yl]-4-ethyl­sulfanyl-1*H*-pyrazole-3-carbo­nitrile, is also an important inter­mediate in the preparation of ethiprole. In view of the importance of phenyl­pyrazoles, especially in the context of their use in insecticides, this paper reports the synthesis, crystal structure, and spectroscopic studies of the phenyl­pyrazole derivative, C_15_H_8_N_4_Cl_2_F_6_OS (**I**).

## 2. Structural commentary

**Figure 1:**
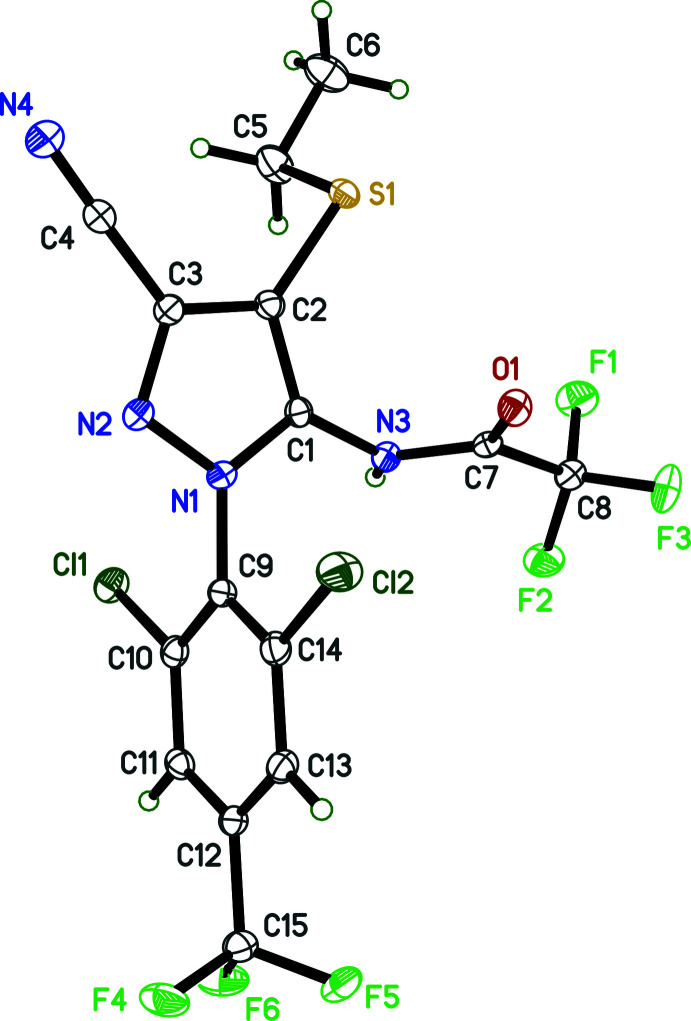
An ellipsoid plot (50% probability) of **I**.

The mol­ecular structure of **I** (Fig. 1 ▸), consists of a pyrazole ring with four chemically diverse substituents. A 2,6-di­chloro-4-tri­fluoro­methyl­phenyl group is attached to atom N1 of the pyrazole ring. A 2,2,2-tri­fluoro­acetamide group is attached to the adjacent carbon of the pyrazole, with ethyl­sulfanyl and cyano substituents attached sequentially at the next two carbon atoms of the pyrazole. The pyrazole and phenyl rings are essentially perpendicular, forming a dihedral angle of 89.80 (5)°. The mean plane of the amide group (r.m.s. deviation = 0.0079 Å) forms a dihedral angle of 74.33 (6)° with the pyrazole ring, while the dihedral angle between the plane of the ethyl­sulfanyl substituent and the pyrazole is 81.31 (8)°. There are no unusual bond lengths, bond angles, or torsion angles in the structure, and no noteworthy intra­molecular inter­actions.

## 3. Supra­molecular features

**Figure 2:**
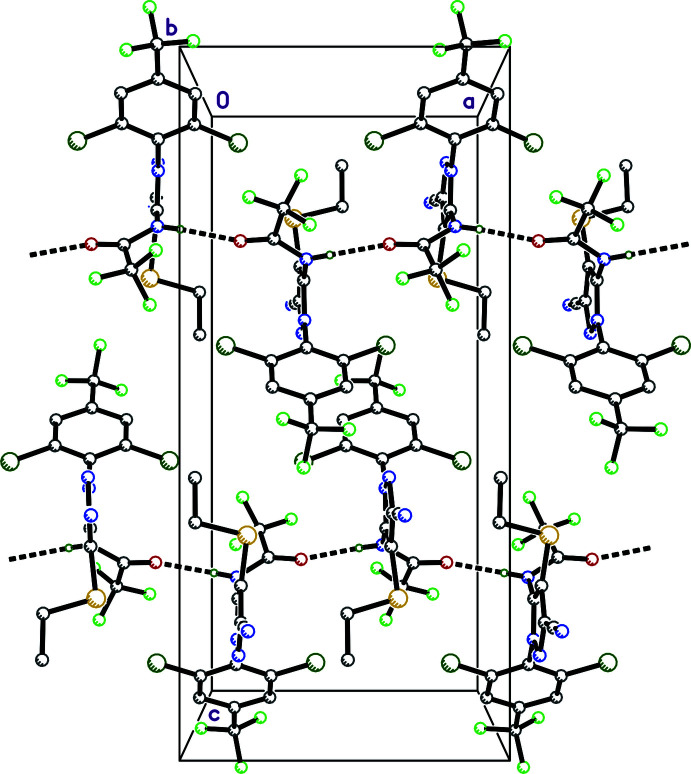
A packing plot of **I** showing strong hydrogen-bonded chains (thick dashed lines) along the *a*-axis direction.

**Figure 3:**
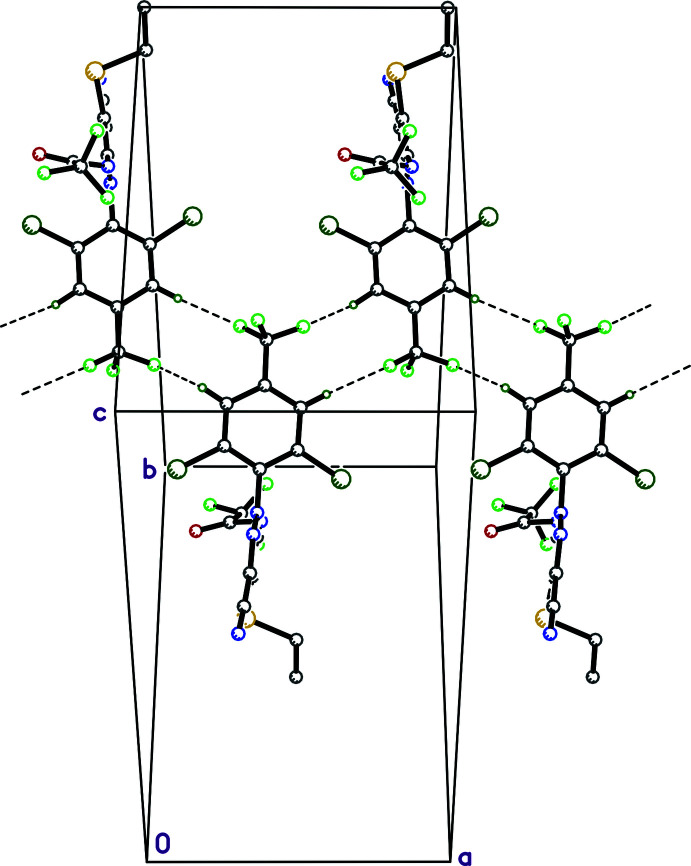
A partial packing plot of **I** showing zigzag chains along the *a*-axis direction resulting from weak C—H⋯F contacts (thin dashed lines).

**Figure 4:**
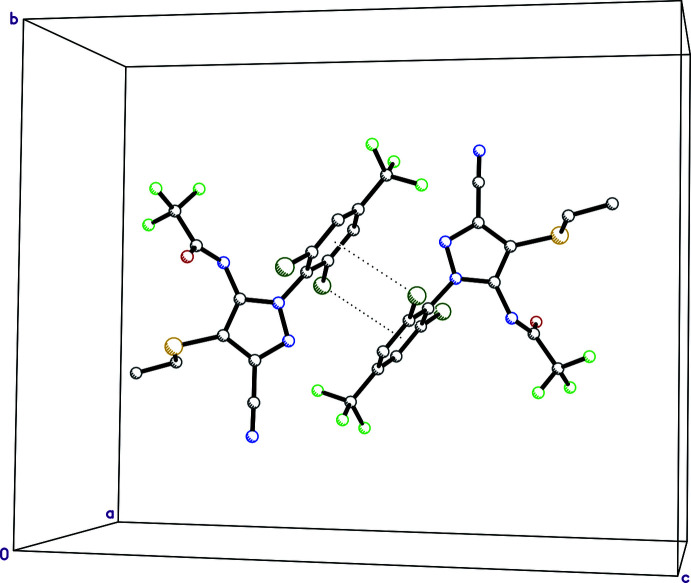
Pairs of inversion-related mol­ecules in **I** showing mutual contacts between Cl and the benzene rings (dotted lines).

**Figure 5:**
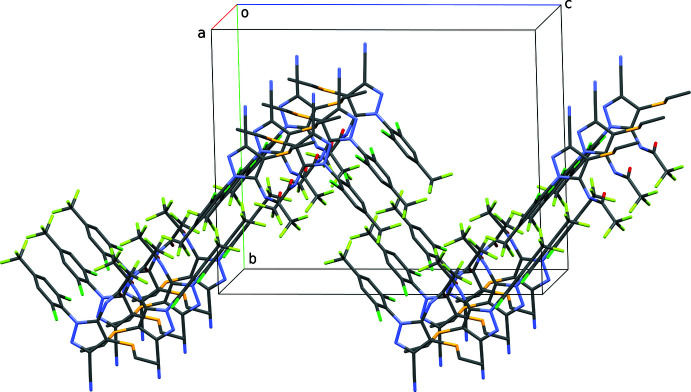
A partial packing plot of **I** showing pleated sheets that extend in the *ac* plane. Diagram generated using *Mercury* (Macrae *et al.*, 2020 ▸).

There is only one strong inter­molecular hydrogen bond in **I**, namely N3—H3*N*⋯O1^i^ (symmetry codes as per Table 1 ▸), between *c*-glide related acetamide groups (Table 1 ▸), which propagates to form chains that extend parallel to the *a*-axis (Fig. 2 ▸). The default HTAB command in *SHELXL* (Sheldrick, 2015*b* ▸) also flags three C—H⋯F close contacts (Table 1 ▸). Two of these, C11—H11⋯F5^iii^ and C13—H13⋯F6^iv^, are oriented so as to associate 2_1_-screw-related mol­ecules into chains, which again extend parallel to the *a*-axis (Fig. 3 ▸). There are no π–π stacking inter­actions, but inversion-related mol­ecules have their Cl1 atoms mutually located directly over the benzene rings of their inversion-related counterparts [Cl1⋯*Cg*(C9–C14)^v^ = 3.4967 (6) Å, where *Cg* represents the ring centroid], as shown in Table 1 ▸ and Fig. 4 ▸. These combine to produce pleated sheets that extend in the *ac* plane (Fig. 5 ▸), which then stack along the *b*-axis direction. Atom–atom contact coverages derived from a Hirshfeld-surface analysis using *CrystalExplorer* (Spackman *et al.*, 2021 ▸) are given in Table 2 ▸.

## 4. Database survey

A search of the Cambridge Structural Database (CSD version 5.43 with updates through June 2022; Groom *et al.*, 2016 ▸) for the 1-phenyl-cyano­pyrazole fragment of **I** gave 82 hits. A search on this fragment with any nitro­gen-bound substituent at the equivalent of C1 (*i.e.*, the carbon adjacent to the substituted nitro­gen) gave 76 hits, and a subsequent search with 2,6-di­chloro-4-(tri­fluoro­meth­yl)phenyl attached at N1 of the pyrazole ring gave 60 hits. Further addition of any sulfur-bound substituent at the equivalent of C2 gave nine hits, only eight of which are unique. Two of these structures, FOCCUW (Tang, Zhong, Li *et al.*, 2005 ▸) and TOLFUY (Du *et al.*, 2019 ▸) are dimers. The remaining six, along with three other similar structures, are listed in Table 3 ▸.

## 5. Synthesis, crystallization and spectroscopic details

**Figure 6:**
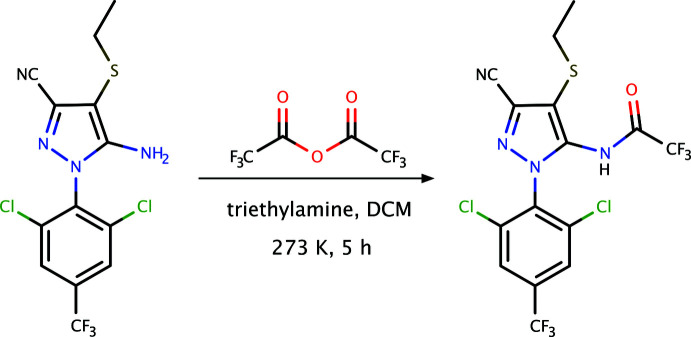
The overall reaction scheme for the synthesis of **I**.

Tri­fluoro­acetic anhydride (550 µL, 3.8 mmol) was added dropwise to a stirred solution of 5-amino-1-[2,6-di­chloro-4-(tri­fluoro­meth­yl)phen­yl]-4-ethyl­sulfanyl-1*H*-pyrazole-3-carb­o­nitrile (a gift from Honeychem Pharma: 724 mg, 1.9 mmol), tri­ethyl­amine (412 mg, 5.7 mmol) and DCM (5 ml) at 273 K. The reaction was kept at 273 K for 5 h, warmed to room temperature over 3 h, quenched with water and extracted with DCM three times. An overall scheme for the reaction is shown in Fig. 6 ▸. The combined organic extracts were washed with water and brine. The crude residue obtained after drying with sodium sulfate followed by concentration, was purified by column chromatography using ethyl acetate:hexane (2:3) as eluent to give *N*-{3-cyano-1-[2,6-di­chloro-4-(tri­fluoro­meth­yl)phen­yl]-4-(ethyl­sulfan­yl)-1*H*-pyrazol-5-yl}-2,2,2-tri­fluoro­acetamide (C_15_H_8_Cl_2_F_6_N_4_OS, **I**, yield = 600 mg, 85%).

The product was dissolved in ethanol at 333 K and stirred for 30 min. The resulting solution was allowed to cool slowly to room temperature with slow evaporation. X-ray-quality crystals appeared in two days (m.p. 366–367 K).

The title compound was characterized by IR and ^1^H NMR spectroscopies, as follows: FT–IR (ν in cm^−1^): 3227 (N—H stretching), 2250 (C=N stretching), 1737 (C=O stretching), 1694–1652 (C=C stretching), 1313, 1222 (C—F stretching), 881, 818 (*s*, Ar–C—H bending), 711, 628 (C—Cl). ^1^H NMR: DMSO–*d*_6_ (400 MHz, δ ppm): 12.42 (*b*, 1H, NH), 8.36 (*s*, 2H, Ar—H), 2.90–2.85 (*q*, 2H, CH_2_, *J* = 7.6 Hz), 1.19–1.15 (*t*, 3H, CH_3_, *J* = 7.6 Hz).

## 6. Refinement

Crystal data, data collection, and structure refinement details are summarized in Table 4 ▸. All H atoms were found in difference-Fourier maps. Carbon-bound hydrogens were subsequently included in the refinement using riding models, with constrained distances set to 0.98 Å (*R*CH_3_), 0.99 Å (*R*_2_CH_2_) and 0.95 Å (*R*_2_CH). The nitro­gen-bound hydrogen-atom coordinates were refined freely. *U*_iso_(H) parameters were set to values of either 1.2*U*_eq_ or 1.5*U*_eq_ (*R*CH_3_ only) of the attached atom.

## Supplementary Material

Crystal structure: contains datablock(s) I, global. DOI: 10.1107/S2056989022009653/tx2059sup1.cif

Structure factors: contains datablock(s) I. DOI: 10.1107/S2056989022009653/tx2059Isup2.hkl

CCDC reference: 2210523

Additional supporting information:  crystallographic information; 3D view; checkCIF report

## Figures and Tables

**Table 1:** Hydrogen bonds and other short contacts (Å, °) in **I** *Cg*(C9–C14) represents the centroid of C9–C14 benzene ring.

Atoms	*D*—H	H⋯*A*	*D*⋯*A*	*D*—H⋯*A*
N3—H3*N*⋯O1^i^	0.855 (16)	2.034 (16)	2.8172 (13)	151.9 (14)
C5—H5*B*⋯F2^ii^	0.99	2.58	3.5641 (16)	173.9
C11—H11⋯F5^iii^	0.95	2.62	3.4873 (15)	151.8
C13—H13⋯F6^iv^	0.95	2.39	3.2071 (15)	143.8
Cl1⋯*Cg*(C9–C14)^v^			3.4967 (6)	

Symmetry codes: (i) *x* + 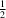 , *y*, −*z* + 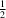 ; (ii) −*x* + 1, *y* − 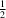 , −*z* + 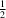 ; (iii) *x* + 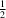 , −*y* + 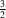 , −*z* + 1; (iv) *x* − 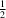 , −*y* + 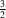 , −*z* + 1; (v) −*x* + 1, −*y* + 1, −*z* + 1.

**Table 2:** Atom–atom contact coverages (%) in **I**

Atom contacts	%	Atom contacts	%
H⋯F/F⋯H	23.0	F⋯Cl/Cl⋯F	8.3
N⋯F/F⋯N	7.3	C⋯H/H⋯C	7.1
H⋯Cl/Cl⋯H	7.1	H⋯N/N⋯H	6.9
H⋯O/O⋯H	5.9	H⋯H	4.8
C⋯F/F⋯C	3.8	C⋯Cl/Cl⋯C	3.8
C⋯N/N⋯C	3.4	F⋯S/S⋯F	3.0
S⋯Cl/Cl⋯S	1.9	Cl⋯Cl	1.3
H⋯S/S⋯H	1.3	O⋯Cl/Cl⋯O	1.2
C⋯C	0.9	O⋯N/N⋯O	0.8
N⋯Cl/Cl⋯N	0.7	N⋯N	0.3
O⋯F/F⋯O	0.2	C⋯S/S⋯C	0.2
C⋯O/O⋯C	0.1		

All other atom–atom contact coverages are ∼0.0%

**Table 3:** Some structures similar to **I** deposited in the CSD All entries have 2,6-di­chloro-4-(tri­fluoro­meth­yl)phenyl and cyano groups attached at the equivalent of N1 and C3 of **I**, respectively. Substituents *R*′ and *R* represent groups attached at the equivalent of C1 and C2 in **I**, respectively.

CSD code	*R*′	*R*"	Reference
DUKVAJ	NHCOCH_2_Ph	SOCF_3_	Chen *et al.* (2020 ▸)
EFIXEZ	NHCOCHCHPh	SOCF_3_	Chen (2019 ▸)
PAZFAY	NH_2_	SCF_3_	Tang, Zhong, Lin *et al.* (2005 ▸)
TOLFAE	NHCH_2_PhOMe	SOCF_3_	Chen & Wu (2019 ▸)
YEGJAY	NH_2_	SOCF_3_	Park *et al.* (2017 ▸)
ZITNAU	NHCHPhF	SOCF_3_	Chen *et al.* (2019 ▸)
GIXDAT	NH_2_	I	Li *et al.* (2007 ▸)
HILTUS	NH_2_	H	Luo *et al.* (2007 ▸)
TIDNUP	NH_2_	CF_3_	Hainzl & Casida (1996 ▸)

**Table 4:** Experimental details

Crystal data	
Chemical formula	C_15_H_8_Cl_2_F_6_N_4_OS
*M*_r_	477.21
Crystal system, space group	Orthorhombic, *P* *b* *c* *a*
Temperature (K)	90
*a*, *b*, *c* (Å)	9.9350 (3), 17.5133 (7), 21.4662 (8)
*V* (Å^3^)	3735.0 (2)
*Z*	8
Radiation type	Mo *K*α
μ (mm^−1^)	0.53
Crystal size (mm)	0.30 × 0.23 × 0.19

Data collection	
Diffractometer	Bruker D8 Venture dual source
Absorption correction	Multi-scan (*SADABS*; Krause *et al.*, 2015 ▸)
*T*_min_, *T*_max_	0.831, 0.958
No. of measured, independent and observed [*I* > 2σ(*I*)] reflections	27352, 4271, 3893
*R*_int_	0.036
(sin θ/λ)_max_ (Å^−1^)	0.650

Refinement	
*R*[*F* ^2^ > 2σ(*F* ^2^)], *wR*(*F* ^2^), *S*	0.026, 0.064, 1.04
No. of reflections	4271
No. of parameters	267
H-atom treatment	H atoms treated by a mixture of independent and constrained refinement
Δρ_max_, Δρ_min_ (e Å^−3^)	0.42, −0.25

Computer programs: *APEX3* (Bruker, 2016 ▸), *SHELXT* (Sheldrick, 2015*a* ▸), *SHELXL2019/2* (Sheldrick, 2015*b* ▸), *XP* in *SHELXTL* (Sheldrick, 2008 ▸), and *publCIF* (Westrip, 2010 ▸).

## supplementary crystallographic information

### Crystal data

**Table:**

C_15_H_8_Cl_2_F_6_N_4_OS	*D*_x_ = 1.697 Mg m^−^^3^
*M**_r_* = 477.21	Mo *K*α radiation, λ = 0.71073 Å
Orthorhombic, *P**b**c**a*	Cell parameters from 9972 reflections
*a* = 9.9350 (3) Å	θ = 2.5–27.5°
*b* = 17.5133 (7) Å	µ = 0.53 mm^−^^1^
*c* = 21.4662 (8) Å	*T* = 90 K
*V* = 3735.0 (2) Å^3^	Cut block, colourless
*Z* = 8	0.30 × 0.23 × 0.19 mm
*F*(000) = 1904	

### Data collection

**Table:**

Bruker D8 Venture dual source diffractometer	4271 independent reflections
Radiation source: microsource	3893 reflections with *I* > 2σ(*I*)
Detector resolution: 7.41 pixels mm^-1^	*R*_int_ = 0.036
φ and ω scans	θ_max_ = 27.5°, θ_min_ = 2.3°
Absorption correction: multi-scan (*SADABS*; Krause *et al.*, 2015)	*h* = −12→10
*T*_min_ = 0.831, *T*_max_ = 0.958	*k* = −22→22
27352 measured reflections	*l* = −27→27

### Refinement

**Table:**

Refinement on *F*^2^	Secondary atom site location: difference Fourier map
Least-squares matrix: full	Hydrogen site location: mixed
*R*[*F*^2^ > 2σ(*F*^2^)] = 0.026	H atoms treated by a mixture of independent and constrained refinement
*wR*(*F*^2^) = 0.064	*w* = 1/[σ^2^(*F*_o_^2^) + (0.0273*P*)^2^ + 2.0142*P*] where *P* = (*F*_o_^2^ + 2*F*_c_^2^)/3
*S* = 1.04	(Δ/σ)_max_ = 0.001
4271 reflections	Δρ_max_ = 0.42 e Å^−^^3^
267 parameters	Δρ_min_ = −0.25 e Å^−^^3^
0 restraints	Extinction correction: SHELXL-2019/2 (Sheldrick 2015b), Fc^*^=kFc[1+0.001xFc^2^λ^3^/sin(2θ)]^-1/4^
Primary atom site location: structure-invariant direct methods	Extinction coefficient: 0.0017 (3)

### Special details

**Table:**

Geometry. All esds (except the esd in the dihedral angle between two l.s. planes) are estimated using the full covariance matrix. The cell esds are taken into account individually in the estimation of esds in distances, angles and torsion angles; correlations between esds in cell parameters are only used when they are defined by crystal symmetry. An approximate (isotropic) treatment of cell esds is used for estimating esds involving l.s. planes.

### Fractional atomic coordinates and isotropic or equivalent isotropic displacement parameters (Å^2^)

**Table:**

	*x*	*y*	*z*	*U*_iso_*/*U*_eq_	
Cl1	0.63935 (3)	0.51208 (2)	0.40861 (2)	0.01864 (8)	
Cl2	0.09945 (3)	0.53537 (2)	0.40975 (2)	0.02419 (9)	
S1	0.32287 (3)	0.38515 (2)	0.20184 (2)	0.01658 (8)	
F1	0.35596 (9)	0.62440 (5)	0.15112 (4)	0.02447 (19)	
F2	0.38608 (8)	0.69639 (4)	0.23078 (4)	0.02336 (18)	
F3	0.18898 (8)	0.68747 (5)	0.18872 (4)	0.02659 (19)	
F4	0.36645 (9)	0.70022 (5)	0.59601 (4)	0.02648 (19)	
F5	0.30334 (10)	0.77508 (5)	0.52258 (4)	0.0323 (2)	
F6	0.51380 (8)	0.75448 (5)	0.53817 (4)	0.02669 (19)	
O1	0.15013 (9)	0.55566 (5)	0.24758 (4)	0.01724 (19)	
N1	0.36297 (11)	0.47239 (6)	0.36809 (5)	0.0145 (2)	
N2	0.35250 (11)	0.39898 (6)	0.38684 (5)	0.0168 (2)	
N3	0.37119 (10)	0.55065 (6)	0.27566 (5)	0.0130 (2)	
H3N	0.4502 (17)	0.5686 (8)	0.2699 (7)	0.016*	
N4	0.30803 (13)	0.21428 (6)	0.33539 (6)	0.0255 (3)	
C1	0.36015 (12)	0.47954 (7)	0.30511 (6)	0.0131 (2)	
C2	0.34539 (12)	0.40781 (7)	0.28031 (6)	0.0141 (2)	
C3	0.34127 (13)	0.36032 (7)	0.33357 (6)	0.0155 (2)	
C4	0.32423 (14)	0.27879 (7)	0.33502 (6)	0.0185 (3)	
C5	0.49124 (14)	0.35383 (9)	0.18065 (6)	0.0234 (3)	
H5A	0.556533	0.395746	0.187298	0.028*	
H5B	0.518388	0.309910	0.206827	0.028*	
C6	0.48964 (15)	0.33069 (9)	0.11230 (7)	0.0275 (3)	
H6A	0.460330	0.374098	0.086883	0.041*	
H6B	0.427263	0.287945	0.106458	0.041*	
H6C	0.580318	0.315148	0.099566	0.041*	
C7	0.26356 (12)	0.58029 (6)	0.24545 (5)	0.0131 (2)	
C8	0.29906 (13)	0.64906 (7)	0.20371 (6)	0.0166 (3)	
C9	0.37120 (13)	0.53275 (7)	0.41254 (6)	0.0141 (2)	
C10	0.49581 (12)	0.55813 (7)	0.43330 (6)	0.0140 (2)	
C11	0.50542 (12)	0.61972 (7)	0.47388 (5)	0.0145 (2)	
H11	0.590543	0.637395	0.487965	0.017*	
C12	0.38740 (13)	0.65467 (7)	0.49325 (6)	0.0143 (2)	
C13	0.26172 (13)	0.62922 (7)	0.47417 (6)	0.0160 (2)	
H13	0.182136	0.653612	0.488522	0.019*	
C14	0.25432 (13)	0.56765 (7)	0.43387 (6)	0.0156 (2)	
C15	0.39317 (13)	0.72121 (7)	0.53760 (6)	0.0176 (3)	

### Atomic displacement parameters (Å^2^)

**Table:**

	*U*^11^	*U*^22^	*U*^33^	*U*^12^	*U*^13^	*U*^23^
Cl1	0.01593 (15)	0.01905 (15)	0.02095 (16)	0.00316 (11)	0.00349 (11)	−0.00045 (11)
Cl2	0.01509 (16)	0.02479 (17)	0.03269 (19)	−0.00092 (12)	−0.00540 (13)	−0.00730 (13)
S1	0.01774 (16)	0.01755 (15)	0.01445 (15)	0.00235 (11)	−0.00419 (12)	−0.00360 (11)
F1	0.0328 (5)	0.0231 (4)	0.0175 (4)	−0.0014 (3)	0.0079 (3)	−0.0003 (3)
F2	0.0286 (4)	0.0160 (4)	0.0255 (4)	−0.0086 (3)	−0.0006 (3)	−0.0001 (3)
F3	0.0246 (4)	0.0215 (4)	0.0336 (5)	0.0074 (3)	−0.0018 (4)	0.0101 (3)
F4	0.0389 (5)	0.0253 (4)	0.0152 (4)	−0.0056 (4)	0.0072 (3)	−0.0047 (3)
F5	0.0409 (5)	0.0182 (4)	0.0378 (5)	0.0121 (4)	−0.0129 (4)	−0.0102 (4)
F6	0.0286 (4)	0.0231 (4)	0.0285 (4)	−0.0110 (3)	0.0052 (4)	−0.0093 (3)
O1	0.0122 (4)	0.0175 (4)	0.0220 (5)	−0.0002 (3)	−0.0008 (4)	0.0010 (4)
N1	0.0183 (5)	0.0108 (5)	0.0143 (5)	0.0006 (4)	−0.0015 (4)	−0.0007 (4)
N2	0.0213 (5)	0.0118 (5)	0.0172 (5)	0.0005 (4)	−0.0025 (4)	0.0006 (4)
N3	0.0104 (5)	0.0123 (5)	0.0162 (5)	−0.0011 (4)	−0.0003 (4)	0.0007 (4)
N4	0.0355 (7)	0.0170 (5)	0.0239 (6)	−0.0005 (5)	−0.0053 (5)	0.0002 (4)
C1	0.0109 (5)	0.0138 (5)	0.0146 (6)	0.0012 (4)	−0.0017 (4)	−0.0001 (4)
C2	0.0131 (5)	0.0145 (6)	0.0147 (6)	0.0012 (4)	−0.0017 (5)	−0.0018 (4)
C3	0.0164 (6)	0.0132 (5)	0.0168 (6)	0.0008 (5)	−0.0026 (5)	−0.0006 (4)
C4	0.0230 (7)	0.0172 (6)	0.0153 (6)	0.0009 (5)	−0.0040 (5)	−0.0005 (5)
C5	0.0188 (6)	0.0332 (7)	0.0181 (6)	0.0040 (6)	0.0001 (5)	−0.0035 (5)
C6	0.0248 (7)	0.0388 (8)	0.0190 (7)	−0.0018 (6)	0.0022 (6)	−0.0067 (6)
C7	0.0145 (5)	0.0116 (5)	0.0132 (5)	0.0018 (4)	0.0006 (5)	−0.0026 (4)
C8	0.0180 (6)	0.0142 (6)	0.0177 (6)	0.0007 (5)	−0.0001 (5)	−0.0002 (5)
C9	0.0200 (6)	0.0110 (5)	0.0115 (6)	−0.0001 (4)	−0.0017 (5)	0.0004 (4)
C10	0.0150 (6)	0.0139 (5)	0.0131 (6)	0.0013 (4)	0.0014 (5)	0.0024 (4)
C11	0.0161 (6)	0.0144 (5)	0.0132 (6)	−0.0024 (4)	−0.0014 (5)	0.0016 (4)
C12	0.0195 (6)	0.0114 (5)	0.0120 (5)	0.0000 (4)	−0.0002 (5)	0.0013 (4)
C13	0.0169 (6)	0.0144 (5)	0.0166 (6)	0.0029 (5)	0.0003 (5)	0.0003 (4)
C14	0.0151 (6)	0.0150 (5)	0.0166 (6)	−0.0003 (5)	−0.0027 (5)	0.0014 (5)
C15	0.0209 (6)	0.0146 (6)	0.0173 (6)	−0.0005 (5)	0.0000 (5)	−0.0012 (5)

### Geometric parameters (Å, º)

**Table:**

Cl1—C10	1.7219 (12)	C1—C2	1.3722 (16)
Cl2—C14	1.7191 (13)	C2—C3	1.4143 (17)
S1—C2	1.7450 (13)	C3—C4	1.4381 (17)
S1—C5	1.8181 (14)	C5—C6	1.5222 (19)
F1—C8	1.3342 (15)	C5—H5A	0.9900
F2—C8	1.3312 (15)	C5—H5B	0.9900
F3—C8	1.3237 (15)	C6—H6A	0.9800
F4—C15	1.3333 (15)	C6—H6B	0.9800
F5—C15	1.3381 (15)	C6—H6C	0.9800
F6—C15	1.3327 (15)	C7—C8	1.5421 (16)
O1—C7	1.2075 (15)	C9—C10	1.3888 (17)
N1—N2	1.3511 (14)	C9—C14	1.3898 (17)
N1—C1	1.3581 (16)	C10—C11	1.3898 (17)
N1—C9	1.4264 (15)	C11—C12	1.3865 (17)
N2—C3	1.3337 (16)	C11—H11	0.9500
N3—C7	1.3540 (16)	C12—C13	1.3877 (18)
N3—C1	1.4010 (15)	C12—C15	1.5059 (17)
N3—H3N	0.855 (16)	C13—C14	1.3843 (17)
N4—C4	1.1412 (17)	C13—H13	0.9500
			
C2—S1—C5	101.08 (6)	N3—C7—C8	113.4 (1)
N2—N1—C1	112.5 (1)	F3—C8—F2	109.04 (10)
N2—N1—C9	120.69 (10)	F3—C8—F1	108.0 (1)
C1—N1—C9	126.78 (10)	F2—C8—F1	107.2 (1)
C3—N2—N1	103.54 (10)	F3—C8—C7	110.44 (10)
C7—N3—C1	119.68 (10)	F2—C8—C7	112.41 (10)
C7—N3—H3N	121 (1)	F1—C8—C7	109.61 (10)
C1—N3—H3N	117.7 (10)	C10—C9—C14	119.89 (11)
N1—C1—C2	107.7 (1)	C10—C9—N1	120.19 (11)
N1—C1—N3	121.98 (10)	C14—C9—N1	119.90 (11)
C2—C1—N3	130.32 (12)	C9—C10—C11	120.71 (11)
C1—C2—C3	103.17 (11)	C9—C10—Cl1	119.31 (9)
C1—C2—S1	126.61 (10)	C11—C10—Cl1	119.97 (10)
C3—C2—S1	130.01 (9)	C12—C11—C10	118.20 (11)
N2—C3—C2	113.09 (11)	C12—C11—H11	120.9
N2—C3—C4	119.69 (11)	C10—C11—H11	120.9
C2—C3—C4	127.21 (11)	C11—C12—C13	122.05 (11)
N4—C4—C3	178.42 (15)	C11—C12—C15	119.94 (11)
C6—C5—S1	108.17 (10)	C13—C12—C15	118.00 (11)
C6—C5—H5A	110.1	C14—C13—C12	118.84 (12)
S1—C5—H5A	110.1	C14—C13—H13	120.6
C6—C5—H5B	110.1	C12—C13—H13	120.6
S1—C5—H5B	110.1	C13—C14—C9	120.27 (12)
H5A—C5—H5B	108.4	C13—C14—Cl2	119.47 (10)
C5—C6—H6A	109.5	C9—C14—Cl2	120.26 (9)
C5—C6—H6B	109.5	F6—C15—F4	106.92 (11)
H6A—C6—H6B	109.5	F6—C15—F5	107.08 (10)
C5—C6—H6C	109.5	F4—C15—F5	106.75 (11)
H6A—C6—H6C	109.5	F6—C15—C12	112.23 (11)
H6B—C6—H6C	109.5	F4—C15—C12	111.94 (10)
O1—C7—N3	125.59 (11)	F5—C15—C12	111.58 (11)
O1—C7—C8	120.96 (11)		
			
C1—N1—N2—C3	−0.77 (14)	N3—C7—C8—F1	77.52 (13)
C9—N1—N2—C3	177.32 (11)	N2—N1—C9—C10	91.61 (15)
N2—N1—C1—C2	0.79 (14)	C1—N1—C9—C10	−90.59 (15)
C9—N1—C1—C2	−177.16 (11)	N2—N1—C9—C14	−90.12 (14)
N2—N1—C1—N3	−179.23 (11)	C1—N1—C9—C14	87.67 (16)
C9—N1—C1—N3	2.82 (19)	C14—C9—C10—C11	−1.93 (18)
C7—N3—C1—N1	−111.17 (13)	N1—C9—C10—C11	176.34 (11)
C7—N3—C1—C2	68.80 (18)	C14—C9—C10—Cl1	177.67 (9)
N1—C1—C2—C3	−0.45 (13)	N1—C9—C10—Cl1	−4.06 (16)
N3—C1—C2—C3	179.58 (12)	C9—C10—C11—C12	0.37 (18)
N1—C1—C2—S1	174.65 (9)	Cl1—C10—C11—C12	−179.23 (9)
N3—C1—C2—S1	−5.3 (2)	C10—C11—C12—C13	1.06 (18)
C5—S1—C2—C1	101.74 (12)	C10—C11—C12—C15	179.53 (11)
C5—S1—C2—C3	−84.50 (13)	C11—C12—C13—C14	−0.90 (18)
N1—N2—C3—C2	0.47 (14)	C15—C12—C13—C14	−179.39 (11)
N1—N2—C3—C4	−178.34 (12)	C12—C13—C14—C9	−0.69 (18)
C1—C2—C3—N2	−0.02 (14)	C12—C13—C14—Cl2	−179.99 (9)
S1—C2—C3—N2	−174.87 (10)	C10—C9—C14—C13	2.09 (18)
C1—C2—C3—C4	178.68 (13)	N1—C9—C14—C13	−176.18 (11)
S1—C2—C3—C4	3.8 (2)	C10—C9—C14—Cl2	−178.62 (9)
C2—S1—C5—C6	179.48 (10)	N1—C9—C14—Cl2	3.11 (16)
C1—N3—C7—O1	10.00 (18)	C11—C12—C15—F6	20.04 (16)
C1—N3—C7—C8	−167.28 (10)	C13—C12—C15—F6	−161.43 (11)
O1—C7—C8—F3	18.96 (16)	C11—C12—C15—F4	−100.19 (13)
N3—C7—C8—F3	−163.61 (10)	C13—C12—C15—F4	78.33 (14)
O1—C7—C8—F2	140.97 (12)	C11—C12—C15—F5	140.24 (12)
N3—C7—C8—F2	−41.60 (14)	C13—C12—C15—F5	−41.23 (16)
O1—C7—C8—F1	−99.91 (13)		

### Hydrogen-bond geometry (Å, º)

**Table:**

*D*—H···*A*	*D*—H	H···*A*	*D*···*A*	*D*—H···*A*
N3—H3*N*···O1^i^	0.855 (16)	2.034 (16)	2.8172 (13)	151.9 (14)
C5—H5*B*···F2^ii^	0.99	2.58	3.5641 (16)	174
C11—H11···F5^iii^	0.95	2.62	3.4873 (15)	152
C13—H13···F6^iv^	0.95	2.39	3.2071 (15)	144

Symmetry codes: (i) *x*+1/2, *y*, −*z*+1/2; (ii) −*x*+1, *y*−1/2, −*z*+1/2; (iii) *x*+1/2, −*y*+3/2, −*z*+1; (iv) *x*−1/2, −*y*+3/2, −*z*+1.
